# Association of absolute and relative hand grip strength with all-cause mortality among middle-aged and old-aged people

**DOI:** 10.1186/s12877-023-04008-8

**Published:** 2023-05-23

**Authors:** Wonjeong Jeong, Jong Youn Moon, Jae-Hyun Kim

**Affiliations:** 1 Cancer Knowledge & Information Center, National Cancer Control Institute, National Cancer Center, Goyang, Republic of Korea; 2 Department of Preventive Medicine, Gachon University College of Medicine, 38-13, Dokjeom-ro 3beon- gil, Namdong-gu, Incheon, 21565 Republic of Korea; 3 Artificial Intelligence and Big-Data Convergence Center, Gachon University Gil Medical Center, Incheon, Republic of Korea; 4 Department of Health Administration, College of Health Science, Dankook University, Cheonan, 31116 Republic of Korea; 5 Institute for Digital Life Convergence, Dankook University, Cheonan, Republic of Korea

**Keywords:** Grip strength, Absolute hand grip strength, Relative hand grip strength, All-cause mortality

## Abstract

**Objectives:**

This study aimed to examine the association of absolute and relative hand grip strength (HGS) with the risk of all-cause mortality among middle-aged and old-aged people in South Korea. Considering that both absolute HGS and relative HGS could be effective measures, an in-depth investigation is necessary to compare the effects of both measures on mortality.

**Methods:**

Data of 9,102 participants, derived from the Korean Longitudinal Study of Aging from 2006 to 2018, were examined. HGS was divided into two categories: absolute HGS and relative HGS (defined as HGS divided by body mass index). The risk of all-cause mortality was the dependent variable. Cox proportional hazard regression was used to analyze the association between HGS and all-cause mortality.

**Results:**

The average of absolute and relative HGS were 25.6 ± 8.7 kg and 1.1 ± 0.4 kg/BMI, respectively. The all-cause mortality rate decreased by 3.2% as absolute HGS increased by 1 kg (adjusted hazard ratio [HR] = 0.968, 95% CI = 0.958–0.978). An increase in relative HGS by 1 kg/BMI was associated with a 22% reduction in risk of all-cause mortality (adjusted HR = 0.780, 95% CI = 0.634–0.960). Individuals with more than two chronic diseases, there was a decrease in all-cause mortality as absolute HGS increased by 1 kg and relative HGS by 1 kg/BMI (absolute HGS; adjusted HR = 0.97, 95% CI = 0.959–0.982, relative HGS; adjusted HR = 0.483, 95% CI = 0.325–0.718).

**Conclusions:**

Our study findings showed that both absolute and relative HGS were inversely associated with the risk of all-cause mortality; a higher absolute/relative HGS was associated with a lower risk of all-cause mortality. Moreover, these findings highlight the importance of improving HGS to alleviate the burden of adverse health problems.

**Supplementary Information:**

The online version contains supplementary material available at 10.1186/s12877-023-04008-8.

## Introduction

With increasing population aging and life expectancy, it has become important for the elderly to be physically and mentally healthy for the rest of their lives [1]. Despite medical advancements and improved healthcare, there are still unexpected and serious conditions such as sarcopenia [2]. Sarcopenia has been considered a predictor of age-related conditions that might accelerate premature mortality [3]. In addition to inevitable aging, chronic diseases are known to modulate and exacerbate sarcopenia [2]. Sarcopenia and its measures have been found to be associated with multiple adverse health outcomes, including impaired activities of daily living, falls, fractures, and all-cause mortality [4].

Hand grip strength (HGS) has shown prognostic utility in the assessment of healthy aging in older adults [5]. The ability to grip is one of the most important functions of the hand, and this can reflect the overall muscular strength for sarcopenia diagnosis [6]. HGS has been widely used as a proxy measure of overall strength because it is simple, quick, and cost-effective [6, 7]. HGS has been highly associated with various factors such as demographic (age, sex), body construct (height, weight, and hand size), and physical or psychosocial variables [6]. Furthermore, HGS is useful as a predictor of health status, muscular strength, and disability, suggesting that poor grip strength is predictive of increased mortality [7]. To emphasize the importance of resistance exercise in physical activity guidelines, the relationship between grip strength and mortality is underscored [8].

HGS measurement is useful in clinical settings for risk screening [9]. However, the measurement and reporting of absolute HGS is not standardized and is not only used in research but also in clinical settings [9, 10]. As HGS is known to be affected by weight, height, and body mass, a new approach for the diagnosis of sarcopenia using body mass index (BMI) was recently considered [11, 12]. As HGS in relation to BMI was identified as a better predictor for outcomes such as cancer, some studies have shown that relative HGS may be a better indicator for muscle weakness than absolute HGS [13, 14]. Thus, relative HGS may represent muscle strength adjusted for body size, which provides more accurate information for sarcopenic obesity screening [15]. Although absolute HGS is being used in various studies or clinical settings, BMI and HGS are significantly associated, and both should be considered more [16].

Therefore, it is necessary to investigate the association of HGS divided into absolute HGS and relative HGS with all-cause mortality to demonstrate the importance of improving HGS and preventing premature mortality. Considering that both absolute HGS and relative HGS could be effective, an in-depth investigation is necessary to compare the effects of absolute and relative HGS on mortality. The primary purpose of this study is to examine the association between absolute and relative HGS and all-cause mortality, aiming to understand the impact of each HGS on mortality. The secondary purpose of this study is to investigate the association between absolute and relative HGS and all-cause mortality stratified by age, gender, and number of chronic diseases. Those variables were one of the significant factors influencing HGS, and analyzing it by age is important.

## Methods

### Data and study participants

Data were extracted from the Korean Longitudinal Study of Ageing (KLoSA) from 2006 to 2018 (follow-up). The KLoSA is designed to collect nationally representative longitudinal data on Koreans aged 45 years or older living in households, selected by multistage stratified probability sampling. A multistage stratified sampling design was used to select household units according to geographical area, including both urban and rural areas. In case of refusal to participate, another subject was selected from an additional, similar sample from the same district [16, 17]. The KLoSA was designed to help develop policies to address health and social issues that are emerging trends related to population aging. As this study used secondary data, additional individual informed consent was not required. The KLoSA survey data is publicly available, so ethical approval was not applicable for this study. This study did not collect inform consent from the participants, because their information was fully anonymized prior to analysis. All individuals were anonymized and de-identified prior to the analysis. To estimate the association between HGS and mortality among people aged 45 years or older, we included 9,102 participants at baseline in 2006 who had no missing information and followed up until 2018 to determine whether a respondent was in a state in which the measurement could be performed. They were followed up for 13 years for all-cause mortality, and as the KLoSA survey was measured every two years, participants were also assessed every two years.

### Variables

The primary independent variable was HGS, which was performed in a sitting position with the elbow fixed at 90° on both sides and measured using a hand grip dynamometer (model number, NO6103; Manufacturer: Tanita Corp., Tokyo, Japan) [12]. The average of grip strength was calculated on both hands. If the subjects were unable to perform the grip test, the value from the other hand was used for the analysis [18]. Absolute HGS was measured in kilograms(kg). Relative HGS was defined as HGS (kg) divided by BMI (kg/m^2^) and was measured in kg/BMI. Subgroup analysis was conducted stratified into age, gender, and number of chronic diseases. For age, participants were divided into those 65 years of age or older, as age and muscle weakness are significantly related [19, 20]. In Korea, the age of 65 is generally considered the beginning of the old age, as workers typically retire at this age [21]. Additionally, the analyses included variables such as the Center for Epidemiologic Studies Depression scale (CES-D) score, Mini-Mental State Examination (MMSE) score, age, sex, education, smoking status, alcohol consumption, marital status, self-rated health, and number of chronic diseases. The CES-D scale is believed to be a good indicator of depression because of its high sensitivity, specificity, and internal consistency, which inquires whether the ten items were experienced or carried out during the past week [22]. MMSE, which is a global measurement that can quickly and easily measure cognitive functions and has been widely used in clinical evaluation, was used to calculate cognitive decline [23]. The number of chronic diseases was measured based on self-reported responses indicating whether the individual had ever been diagnosed with each disease by a physician. Chronic diseases included hypertension, diabetes, cancer, chronic obstructive pulmonary disease, liver disease, cardiovascular disease, cerebrovascular disease, mental illness, and arthritis.

All-cause mortality during the period from 2006 to the end of follow-up (2018) was the main outcome of the study. Death over a maximum follow-up period of 12 years was determined using death certificates.

Covariates were as follows: age (45–54, 55–64, 65–74 and ≥ 75 years), sex (male and female), education (elementary, middle, high school, and ≥ college), smoking status (non-smoker, former smoker, and smoker), alcohol consumption (never, former drinker, and drinker), marital status (married and single [including separated, divorced]), self-rated health (very good, good, moderate, bad, and very bad), and comorbidities such as hypertension, diabetes, cancer, chronic obstructive pulmonary disease, liver disease, heart disease, cerebrovascular diseases, mental illness, and arthritis or rheumatoid arthritis (0, 1, 2, and ≥ 3). Participants’ smoking status was categorized based on a self-reported question that asked whether they currently smoke, have smoked in the past, or have never smoked. Similarly, alcohol consumption was also determined based on self-reported questions that asked whether they usually consume alcohol.

### Statistical analysis

Chi-square tests were performed to investigate the general characteristics of the study population. The Cox proportional hazards model was used to analyze the association between HGS and all-cause mortality, determined using the adjusted hazard ratio (HR) and 95% confidence interval (CI). Survival time was defined as the number of days from the date of HGS measurement to the date of death or December 31, 2018, whichever occurred first. The Akaike Information Criteria (AIC) were used for model selection, in which that a lower value indicates a better model. Differences were considered statistically significant at P < 0.05. Kaplan-Meier was used to evaluate the time taken to all-cause mortality to HGS that affected the time interval. Cumulative incidence of all-cause mortality was calculated by the product limit (Kaplan-Meier) method of survival probability. All data analyses were performed using the SAS software (version 9.4; SAS Institute Inc., Cary, NC, USA).

### Ethics Declaration

All procedures performed in studies involving human participants were in accordance with the ethical standards of the institutional and/or national research committee and with the Declaration of Helsinki (1964) and its later amendments or comparable ethical standards. The KLoSA study was approved by the National Statistical Office (Approval number: 33,602), Institutional Review Board of Korea National Institute for Ethics Policy (P01–201,909–22 − 002). The survey was conducted after acquiring verbal consent of the participants by the trained study interviewer. Since the KLoSA database has been released to the public for scientific use, additional ethical approval was not required for this study.

## Results

Table 1 shows the general characteristics of the study population. Among the 9,102 participants, the average of absolute HGS was 25.6 ± 8.7 points and the average of relative HGS was 1.1 ± 0.4 points. Among them, 1,497 (16.45%) were reported to have died by the end of the follow-up period. The average of CES-D score across all groups was 2.8 ± 2.6 points, which was lower in those who survived (Average = 2.7, SD = 2.5) than in those who died (Average = 3.6, SD = 2.9). The average of MMSE score across all groups was 26.1 ± 4.5, which was lower in those who survived (Average = 26.6, SD = 4.0) than in those who died (Average = 23.5, SD = 5.9).

**Table 1:** General characteristics of participants at baseline

	Total			Death					P-value
				No			Yes		
Absolute grip strength (kg)*	25.6	8.7		26.0	8.6		23.1	8.5	< 0.0001
Relative grip strength (kg/BMI)*	1.1	0.4		1.1	0.4		1.0	0.4	< 0.0001
CESD (score)*	2.8	2.6		2.7	2.5		3.6	2.9	< 0.0001
MMSE (score)*	26.1	4.5		26.6	4.0		23.5	5.9	< 0.0001
**Age, n (%)**									< 0.0001
45–54	3,157	34.7		3,003	95.1		154	4.9	
55–64	2,567	28.2		2,306	89.8		261	10.2	
65–74	2,329	25.6		1,772	76.1		557	23.9	
≥ 75	1,049	11.5		524	50.0		525	50.1	
**Gender, n (%)**									< 0.0001
Male	4,088	44.9		3,246	79.4		842	20.6	
Female	5,014	55.1		4,359	86.9		655	13.1	
**Education, n (%)**									< 0.0001
≤Elementary	3,979	43.7		3,020	75.9		959	24.1	
Middle school	1,553	17.1		1,368	88.1		185	11.9	
High school	2,572	28.3		2,322	90.3		250	9.7	
≥College	998	11.0		895	89.7		103	10.3	
**Smoking status, n (%)**									< 0.0001
Non-smoker	6,416	70.5		5,509	85.9		907	14.1	
Former smoker	873	9.6		666	76.3		207	23.7	
Smoker	1,813	19.9		1,430	78.9		383	21.1	
**Alcohol consumption, n (%)**									< 0.0001
Nothing	3,621	39.8		3,078	85.0		543	15.0	
Former drinker	567	6.2		404	71.3		163	28.8	
Drinker	4,914	54.0		4,123	83.9		791	16.1	
**Marital status, n (%)**									< 0.0001
Married	7,292	80.1		6,278	86.1		1,014	13.9	
Separated, divorced	1,733	19.0		1,261	72.8		472	27.2	
Single	77	0.9		66	85.7		11	14.3	
**Self-rated health, n (%)**									< 0.0001
Very good	336	3.7		311	92.6		25	7.4	
Good	3,338	36.7		3,013	90.3		325	9.7	
Moderate	2,983	32.8		2,517	84.4		466	15.6	
Bad	2,011	22.1		1,490	74.1		521	25.9	
Very Bad	434	4.8		274	63.1		160	36.9	
Number of chronic diseases**, n (%)									< 0.0001
0	4,885	53.7		4,313	88.3		572	11.7	
1	2,590	28.5		2,106	81.3		484	18.7	
2	1,130	12.4		845	74.8		285	25.2	
≥ 3	497	5.5		341	68.6		156	31.4	
**Total**	9102	100		7605	83.6		1497	16.45	

* Average and standard deviation (SD) were used

**Hypertension, diabetes, cancer, chronic obstructive pulmonary disease, liver disease, cardiovascular disease, cerebrovascular disease, mental illness, arthritis

Table 2 shows the association of absolute HGS and relative HGS with all-cause mortality after controlling for all covariates. All-cause mortality decreased by 3.2% as absolute HGS increased by 1 kg (adjusted HR = 0.968, 95% CI = 0.958–0.978). The increase in relative HGS by 1 kg/BMI was associated with 22% reduction in risk of all-cause mortality (adjusted HR = 0.780, 95% CI = 0.634–0.960). As the MMSE score increased, the risk of all-cause mortality decreased (absolute: adjusted HR = 0.972, 95% CI = 0.961–0.982; relative: adjusted HR = 0.967, 95% CI = 0.957–0.977). Compared to smokers, non-smokers had a lower risk of all-cause mortality (absolute: adjusted HR = 0.664, 95% CI = 0.574–0.768; relative: adjusted HR = 0.661, 95% CI = 0.572–0.765). Those who did not drink alcohol had a lower risk of all-cause mortality than those who drank alcohol (absolute: adjusted HR = 0.827, 95% CI = 0.724–0.945; relative: adjusted HR = 0.820, 95% CI = 0.717–0.936). Individuals with fewer chronic diseases had a lower risk of all-cause mortality than those with more than three chronic diseases.

**Table 2:** Adjusted effect of grip strength on death

	Death								
	HR	95% CI		P-value		HR	95% CI		P-value
**Absolute grip strength**	0.968	0.958	0.978	< 0.0001					
**Relative grip strength**						0.780	0.634	0.960	0.019
**CESD**	1.000	0.979	1.020	0.964		1.004	0.983	1.025	0.736
**MMSE**	0.972	0.961	0.982	< 0.0001		0.967	0.957	0.977	< 0.0001
**Age**									
45–54	0.160	0.128	0.199	< 0.0001		0.137	0.110	0.171	< 0.0001
55–64	0.263	0.221	0.312	< 0.0001		0.236	0.199	0.279	< 0.0001
65–74	0.496	0.436	0.563	< 0.0001		0.472	0.416	0.536	< 0.0001
≥ 75	1.000					1.000			
**Gender**									
Male	3.322	2.772	3.981	< 0.0001		2.697	2.256	3.224	< 0.0001
Female	1.000					1.000			
**Education**									
≤Elementary	1.236	0.990	1.543	0.061		1.276	1.022	1.593	0.032
Middle school	1.071	0.839	1.369	0.581		1.094	0.856	1.398	0.471
High school	1.035	0.822	1.304	0.768		1.043	0.828	1.314	0.723
≥College	1.000					1.000			
**Smoking status**									
Non-smoker	0.664	0.574	0.768	< 0.0001		0.661	0.572	0.765	< 0.0001
Former smoker	0.830	0.698	0.986	0.035		0.824	0.693	0.980	0.029
Smoker	1.000					1.000			
**Alcohol consumption**									
Nothing	0.827	0.724	0.945	0.005		0.820	0.717	0.936	0.003
Former drinker	0.894	0.741	1.078	0.240		0.908	0.753	1.096	0.315
Drinker	1.000					1.000			
**Marital status**									
Married	0.675	0.371	1.228	0.198		0.653	0.359	1.189	0.163
Separated, divorced	0.923	0.503	1.693	0.796		0.891	0.486	1.633	0.708
Single	1.000					1.000			
**Self-rated health**									
Very good	0.513	0.329	0.800	0.003		0.471	0.302	0.735	0.001
Good	0.593	0.471	0.747	< 0.0001		0.547	0.435	0.688	< 0.0001
Moderate	0.637	0.519	0.780	< 0.0001		0.602	0.491	0.737	< 0.0001
Bad	0.779	0.648	0.935	0.008		0.761	0.634	0.915	0.004
Very Bad	1.000					1.000			
Number of chronic diseases*									
0	0.788	0.648	0.958	0.017		0.806	0.662	0.981	0.032
1	0.788	0.654	0.950	0.013		0.804	0.667	0.969	0.022
2	0.930	0.763	1.133	0.471		0.938	0.770	1.144	0.530
≥ 3	1.000					1.000			
**AIC**	**25,427**					**25,463**			

*Hypertension, diabetes, cancer, chronic obstructive pulmonary disease, liver disease, cardiovascular disease, cerebrovascular disease, mental illness, arthritis

Table 3 shows the results of subgroup analysis between absolute HGS and relative HGS with all-cause mortality, stratified into age, gender and number of chronic diseases. For individuals aged over 65 years, all-cause mortality decreases as absolute HGS increased by 1 kg (≥ 65 years; adjusted HR = 0.97, 95% CI = 0.959–0.982, < 65 years; adjusted HR = 0.961, 95% CI = 0.943–0.979). There was a significant association between an increase in absolute HGS and a decrease in all-cause mortality in men, and a significant association was also found in women (Male; adjusted HR = 0.968, 95% CI = 0.956–0.98, Female; adjusted HR = 0.969, 95% CI = 0.951–0.987). Among individuals with more than two chronic diseases, all-cause mortality decreased by 3% as absolute HGS increased by 1 kg (adjusted HR = 0.97, 95% CI = 0.959–0.982). For those with more than two chronic diseases, an increase in relative HGS by 1 kg/BMI was associated with 51.7% reduction in risk of all-cause mortality (adjusted HR = 0.483, 95% CI = 0.325–0.718).

**Table 3:** Subgroup analysis of the adjusted effect of grip strength on death according to age*

	Death										
	< 65						≥ 65				
	HR	95% CI		P-value	AIC		HR	95% CI		P-value	AIC
**Absolute grip strength**	0.961	0.943	0.979	< 0.0001	6,915		0.97	0.959	0.982	< 0.0001	16,745
**Relative grip strength**	0.716	0.485	1.056	0.0923	6,930		0.788	0.614	1.01	0.0596	16,766

*All variables, including CESD, MMSE, age, gender, education, smoking status, alcohol consumption, marital status, self-rated health, and number of chronic diseases, were adjusted

## Discussion

The purpose of this study was to clarify the association of absolute HGS and relative HGS with all-cause mortality to emphasize the importance of improving HGS. Our findings showed that higher absolute/relative HGS was associated with a lower risk of all-cause mortality. Considering that HGS can be used to diagnose sarcopenia, previous studies have shown that muscle strength can be used to predict mortality [24]. This study indicated that not only absolute HGS but also relative HGS had an inverse association with mortality risk, implying that higher HGS was associated with a lower risk of all-cause mortality. Various measures are used to identify sarcopenia; however, this topic is controversial [24]. Compared to other measurement methods that are relatively expensive and complex, HGS test could be a feasible and acceptable method for routine measurements [25].

As HGS is age-dependent, this study attempted to assess the relationship between HGS and all-cause mortality stratified by age. Based on our results, among those aged < 65 years, those with higher absolute HGS had a lower risk of all-cause mortality. These results were also observed in patients aged > 65 years. Previous studies have shown that higher grip strength was apparent in both sexes and in a wide range of age groups, and that average of HGS was significantly lower in patients who died than in those who survived, with ages ranging from 51 to 60 years and 61 to 70 years [26, 27]. Moreover, among middle-aged individuals, maintenance of muscle strength was highly associated with better health outcomes later in life [26]. In addition, HGS is related to lower skeletal muscle mass and peak oxygen uptake, which could lead to adverse health outcomes and mortality [28].

HGS is a useful marker of frailty and an important predictor of mental illness, such as cognitive function decline and depressive symptoms [29]. Poor HGS predicts accelerated dependency and cognitive decline, which is an indicator of physical well-being and social and psychological health [30].

Critically, muscular weakness can indicate age-related health changes, such as cognitive decline, multimorbidity, and this can lead to an increased risk of mortality [31, 32]. Therefore, our study adjusted the CES-D and MMSE scores, which could greatly influence the association between HGS and mortality risk. A study also showed that sarcopenia was highly associated with mortality risk, which might be an end-point of functional decline [33]. In the present study, the high HGS group showed a higher rate of regular exercise or social activity engagement; this could influence their mental illness, which might predict future mortality [34]. Moreover, as HGS is generally related to the muscular system, it is also associate with multimorbidity [35]. In our study, we found that among individuals with more than two chronic diseases, the higher the HGS, the lower the risk of mortality. Therefore, elevated inflammatory markers or physical activity levels may also influence HGS, as it is important for older adults to maintain sufficient muscle mass to counteract the adverse effects of multimorbidity [36].

It should be noted that our study had several limitations. First, although the validity and reliability of some assessments such as the CES-D or MMSE have been tested, this study included self-reported questionnaires, especially for variables such as BMI and self-rated health, which may result in various recall and/or social desirability biases. Second, other measures of physical fitness besides HGS, such as physical activity level, were not included due to a lack of information. However, HGS remains widely used as a proxy for physical functionality, and it is a feasible and acceptable method for routine measurements [25]. Lastly, the severity of health conditions, such as chronic diseases, was not adjusted due to a lack of information.

Despite these limitations, the strengths of the study are as follows: the KLoSA is a South Korean panel study focusing on the elderly, and it has been verified by experts to have statistical validity and national representativeness. Thus, the results can be generalized to the entire Korean population. Moreover, this study was based on a data of more than 10 years; hence, our results may present the long-term association of absolute and relative HGS with the risk of all-cause mortality. Additionally, including the results of both absolute and relative HGS could provide more specific information about the elderly. Both the CES-D and MMSE scores were adjusted in this study, as it is widely known that HGS is highly associated with cognitive function [29].

## Conclusions

The current study found that both absolute HGS and relative HGS were inversely associated with the risk of all-cause mortality. Those with higher HGS had a lower risk of all-cause mortality among both South Korean middle-aged and old-aged people. The risk of all-cause mortality decreased as both absolute and relative HGS increased among individuals with more than two chronic diseases. This shows that, although HGS decreased with age, in a wide range of age distributions, HGS is a useful predictor of health status. Further research on improving HGS, especially for those with low HGS, to prevent adverse health conditions, which might lead to premature mortality, would provide a better understanding of this association. Moreover, this study provides evidence to support policies for physical activity guidelines.

## Electronic supplementary material

Below is the link to the electronic supplementary material.

Supplementary Material 1

## Data Availability

All data used in this study are available at https://survey.keis.or.kr/klosa/klosa01.jsp with the permission of the reasonable request.
