# Supplementary material for: Association of absolute and relative hand grip strength with all-cause mortality among middle-aged and old-aged people
Source: BMC Geriatr. 2023 May 23;23:321. doi: 10.1186/s12877-023-04008-8 (PMC10207794; doi:10.1186/s12877-023-04008-8)
Supplement: Supplementary file 1 — Supplementary Material 1 [file 12877_2023_4008_MOESM1_ESM.docx]

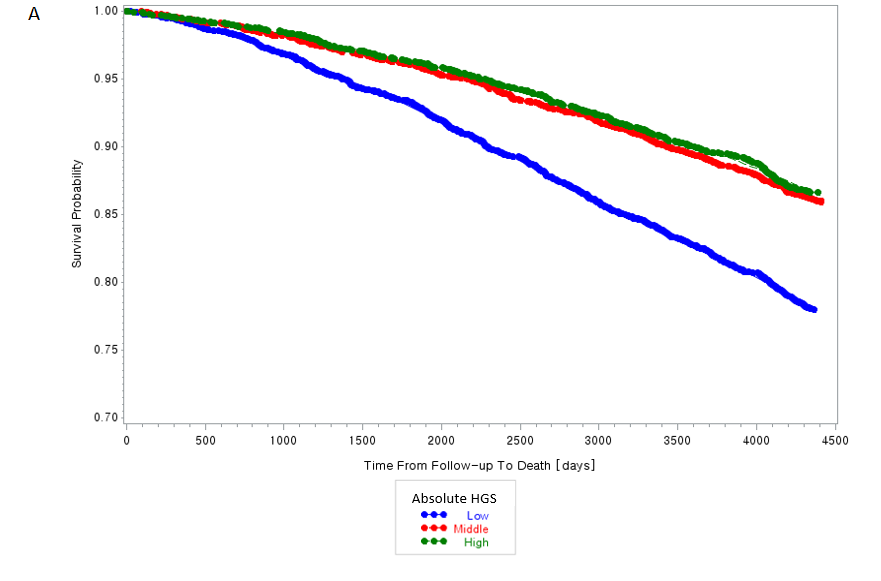


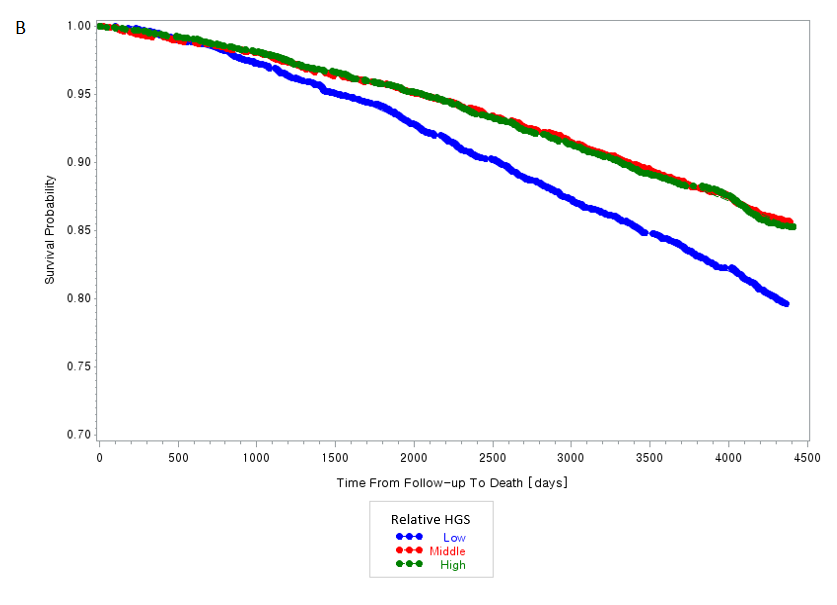


Supplementary 1. Kaplan-Meier curves of all-cause mortality according to handgrip strength. A shows the results of absolute handgrip strength, while B shows the results of relative handgrip strength.
